# Supplementary material for: Diagnosis and management of an inappropriate sinus tachycardia in adolescence based upon a Holter ECG: A retrospective analysis of 479 patients
Source: PLoS One. 2020 Aug 26;15(8):e0238139. doi: 10.1371/journal.pone.0238139 (PMC7449400; doi:10.1371/journal.pone.0238139)
Supplement: S1 Table — (DOCX) [file pone.0238139.s001.docx]

Table: Binary regression analysis Omega-3 fatty acid supplementation (1) versus no Omega-3 fatty acid supplementation (2)

|  | Omega3 | N | Mean | Standard Deviation | Significance |
| --- | --- | --- | --- | --- | --- |
| Age | 1 | 29 | 13,5 | 2,2 | 0,393 |
|  | 2 | 48 | 13,1 | 2,1 | 0,401 |
| Weight Perc | 1 | 29 | 62,9 | 37,3 | 0,881 |
|  | 2 | 48 | 62,5 | 38,0 | 0,880 |
| BMI Perc | 1 | 29 | 64,3 | 37,5 | 0,815 |
|  | 2 | 48 | 63,0 | 39,3 | 0,808 |
| Sys Perc | 1 | 29 | 77,3 | 25,9 | 0,032 |
|  | 2 | 48 | 75,7 | 30,2 | 0,032 |
| Diast Perc | 1 | 29 | 46,6 | 25,4 | 0,730 |
|  | 2 | 47 | 59,9 | 25,9 | 0,728 |
| HF | 1 | 29 | 99,5 | 4,9 | 0,696 |
|  | 2 | 48 | 99,9 | 5,0 | 0,695 |
| SDNN | 1 | 29 | 108,1 | 30,8 | 0,937 |
|  | 2 | 44 | 105,2 | 31,5 | 0,940 |
| RMSSD | 1 | 29 | 22,6 | 7,9 | 0,345 |
|  | 2 | 48 | 22,7 | 6,9 | 0,411 |
| TP 24h | 1 | 27 | 2507,8 | 1661,4 | 0,537 |
|  | 2 | 46 | 2217,3 | 955,6 | 0,567 |
