# Supplementary material for: Diagnosis and management of an inappropriate sinus tachycardia in adolescence based upon a Holter ECG: A retrospective analysis of 479 patients
Source: PLoS One. 2020 Aug 26;15(8):e0238139. doi: 10.1371/journal.pone.0238139 (PMC7449400; doi:10.1371/journal.pone.0238139)
Supplement: S2 Table — (DOCX) [file pone.0238139.s002.docx]

Table: Diagnosis Groups

|  | Congenital Heart Disease | Attention Deficit Disorder | Anorexia Nervsosa | Short Stature | Const. Thinness | Obesity | Hypertension | Pre Hypertension |
| --- | --- | --- | --- | --- | --- | --- | --- | --- |
| Congenital Heart Disease | 55 | 4 | 0 | 11 | 4 | 5 (9.1%) | 10 (18.2%) | 5 |
| Attention Deficit Disorder | x | 86 | 0 | 4 | 4 | 15 (17.4%) | 25 (29.1%) | 10 |
| Anorexia Nervosa | x | x | 34 | 0 | 0 | 0 | 0 | 0 |
| Short Stature | x | x | x | 15 | 1 | 2 (13.3%) | 4 (26.7%) | 0 |
| Constitutional Thinness | x | x | x | x | 20 | 0 | 0 | 0 |
| Obesity | x | x | x | x | x | 130 | 71 (54.6%) | 14 |
| Hypertension | x | x | x | x | x | x | 53 | 0 |
| Pre Hypertension | x | x | x | x | x | x | x | 13 |
|  | 55 | 90 | 34 | 30 | 29 | 152 | 163 | 42 |
| Inapproriate Sinus Tachycardia | 8 (14.5%) | 22 (24.4%) | 1 (2.9%) | 5 (16.7%) | 0 | 23 (15.1%) | 7 (4.3%) | 1 (2.4%) |
| Omega-3-FA | 4 | 8 | 0 | 3 | 0 | 6 | 0 | 1 |
| No Omega-3-FA | 4 | 14 | 1 | 2 | 0 | 17 | 7 | 0 |

| \|  \|  \| \| --- \| --- \| \|  \|  \| \|  \|  \| \|  \|  \| \|  \|  \| \|  \|  \| \|  \|  \| |  |  |  |  |  |  |  |
| --- | --- | --- | --- | --- | --- | --- | --- | --- | --- | --- | --- | --- | --- | --- | --- | --- | --- | --- | --- | --- | --- |
