# Supplementary material for: Diagnosis and management of an inappropriate sinus tachycardia in adolescence based upon a Holter ECG: A retrospective analysis of 479 patients
Source: PLoS One. 2020 Aug 26;15(8):e0238139. doi: 10.1371/journal.pone.0238139 (PMC7449400; doi:10.1371/journal.pone.0238139)
Supplement: Attachment — Submitted filename: Response to Reviewers.odt [file pone.0238139.s003.odt]

Dear Editor,
we are most greatful to  the reviewers for their excellent work. The revision of statistical methods with extend to binary  logistic regression provided new results. This peer reviewing process does improve and sharpen the information that we can learn by analyzing our data set.
 
Reviewer 1 
Reviewer #1: General comments:
This is a retrospective study evaluating 479 children for the association of inappropriate sinus tachycardia (IST, n=79) with heart rate variability (HRV) in Holter-ECG. Healthy controls (n=66) are compared to 8 different groups with attention deficit disorder (ADD, n=86), obesity (n=130), short stature (n=15), congenital heart disease (n=55), anorexia nervosa (n=34), constitutional thinness (n=20), hypertension (n=53) or pre-hypertension (n=13). The IST risk was high in those with ADD, obesity, and short stature and low in those with corrected congenital heart disease and hypertension. IST was associated with a decreased global HRV and hypertension. Omega-3-fatty acid supplementation was associated with decreased HR and increased HRV.

The authors are commended for their elaborate work and data analysis. This is an interesting work, but some issues need to be addressed prior to considering publication in the PLOS ONE Journal.

Specific comments:
1. Methods: Dividing patients into many (not mutually disclosing) groups and separating those with low height (short stature), (constitutional) low or high BMI (obese) and high blood pressure (BP, pre-/hypertension) creates some methodological problems, since further comparisons do not adjust for the differences for such factors. As the authors point out those with obesity and ADD had higher BP, and those with anorexia nervosa had lower BP. Similarly it seems that high BMI/obesity was also present to some extent in other groups too. Please consider using logistic regression to calculate the adjusted risk for IST and report OR and confidence intervals along with p values.

2. Methods: How were the 66 healthy controls chosen? What was the indication for Holter-ECG in these individuals? Consider possible implications/selection bias.

3. Results/Table 1: Please report what is the percentage of obese or hypertensive children in each group and how many children in each group had IST.

4. Methods/Results/Figure 1: Please report how the IST risk was calculated. It seems like the depicted risk represents the percentage/prevalence of IST cases in each group (e.g. 22/86=26% for ADD). However, as described in point 1 this is unadjusted for BP/BMI/height and could be misleading. For example since those with obesity and ADD had high BP, we do not know to what extent BP could bias these findings.

5. Results/Limitations: Omega-3-fatty acid supplementation was up to the patient`s choice. Therefore a selection bias could confound the effect of supplementation. Additionally, since IST has been also associated with psychosocial distress, a strong placebo effect could partially account for the same effect. Please compare baseline characteristics of those who choose supplementation vs. those who did not and comment accordingly in the limitations section.

6. Results/Table 3 & 4: Please report on the sympathetic activity (LF/HF ratio) too. Comment if significant differences become apparent

7. Results/Table 4: Please report the p values (some cells have missing/zero values)

8. Methods/Limitations: HR significantly influences HRV due to both physiological and mathematical reasons. Thus changes in HR should be taken into account when evaluating HRV changes (e.g. obese vs. anorectic children or ADD on/off MPH). This could be implemented in a sensitivity analysis or acknowledged in the limitations section.

Answers to Reviewer 1
Methods: Indeed, dividing our patients into many groups with a broad overlap leads to important methodological problems. We performed a binary logistic regression analysis as proposed by reviewer 1. Heavy work - but a significant improvement of our publication. We deleted Figure 1 due to the misleading interpretation based on IST percentages in the diagnosis groups. We included a new table 5 with the results of the binary logistic regression analysis. The new results according to the Odds ratios are declared in the abstract:
“We found the risk of IST to be high in adolescents with attention deficit disorder (OR=3.5,p<0.001), children with short stature (OR=1.9,p=0.19) and hypertension (OR=2.1,p=0.02); lower in surgically-treated congenital heart disease (OR=1.4,p=0.51) and obesity without hypertension (OR=1.4;p=0.25); and negligible in adolescents with anorexia nervosa (OR=0.3, p=0.26) and constitutional thinness (OR=0.9,p=0.89).
” We explain the collection of the healthy control group and the citation of the published data and the ethic approvement (Reference 8).
 Results: We include a new table with the number and percentage of each diagnosis as supplement to our publication (Tablediagnosisgroups). 
See Point 1
In a new statistic we compare adolescents who received Omega-3-FA with those who received no Omega-3-FA and found no significant group difference. Supplement to our publication (TableOmega3).
The HF/LF ratios are calculated and included in table 3&4.
The p-values are given as <0.001 instead of a zero
See study limitations
Reviewer 2
Reviewer #2: The authors of this manuscript analysed the prevalence of inappropriate sinus tachycardia (IST) in adolescence.
Despite the topic is interesting, the are some critical point:

1) The diagnosis of IST seems to be based only on a single 24-h Holter 12-bit digital ECG. More measurements would have been more appropriate for a better diagnosis. Please report it in the limitation of the study.

2) Any information about the clinic follow-up of these patients has been reported. If it is possible, follow-up data should be included.

3) ECG, echocardiographic or anamnestic information is not be included in the analysis. This is an important limitation for this study.

4) The tables should be improved and integrated with for clinical information of the patients.
Answers to reviewer 2
IST depends on definition based upon the mean 24 hour heart rate >90 bpm or daily heart rates >100 bpm in adults. We adapt this definition to adolescents based upon our normal values. This definition was proofed in each patient.
We focused the follow up on a second Holter ECG according to pharmacologic or nutritional intervention with methylphenidate therapy or omega-3-fatty acid supplementation. That’s a lot for the first big study to inappropriate sinus tachycardia in childhood. Some authors frighten that the current paper is just overwhelmed. Calculating further follow up data would weaken the statistical power.
Nearly all patients had ECG and echocardiographic data to exclude or proof heart defects or cardiomyopathies (see limitations).
We include a supplement (Tablediagnosisgroups) that declare the individual diagnosis and percentages of IST, Obesity, Short Stature and hypertension. The Editor should decide weather to include this data as a 7th table in the original publication.
