# Supplementary material for: Diagnosis and management of an inappropriate sinus tachycardia in adolescence based upon a Holter ECG: A retrospective analysis of 479 patients
Source: PLoS One. 2020 Aug 26;15(8):e0238139. doi: 10.1371/journal.pone.0238139 (PMC7449400; doi:10.1371/journal.pone.0238139)
Supplement: Attachment — Submitted filename: rebuttalletter.docx [file pone.0238139.s004.docx]

Dear Editor,

Thank you again for the careful review of our manuscript.

The specific comments of Reviewer#1 are included in our revision:

**1.**

Indeed, the Odds ratios are not significantly elevated in the kids with short stature, congenital heart disease and obesity without hypertension – we correct the text. Hypertension and prehypertension are significantly more common in kids with inappropriate sinus tachycardia. This fact is in good accordance with data from adults (1) and underline that inappropriate sinus tachycardia is an important cardiovascular risk factor.

**2.**

We proof this table and the statistics that is part of the supplement and not the original publication. There is only a slight difference in the diastolic blood pressure percentile. Furthermore, we demonstrate the absolute blood pressure values and there is only a difference of 2.9 mmHg systolic blood pressure and 5.2 mmHg diastolic blood pressure. I declare this difference in the publication, but I cannot imagine that this is a real bias. I improve this table and delete the two tailed p-values.

Children who didn’t receive omega-3-fatty acids had no follow-up Holter ECG, except those who need a pharmacotherapy.

**Table: Omega-3 Supplementation versus no Omega-3-FA Supplementation**

|  | **Omega-3 Supplementation (N=29)** | **No Omega-3-Supplementation (N=48)** | **Significance** |
| --- | --- | --- | --- |
| **Age [years]** | 13.5 ± 2.2 | 13,1 ± 2.1 | 0,393 |
| **Height Perc [%]** | 44,2 ± 26.7 | 47.2 ± 31.4 | 0,679 |
| **Weight Perc [%]** | 62,9 ± 37,3 | 62,5 ± 38.0 | 0,881 |
| **BMI Perc [%]** | 64,3 ± 37,5 | 63,0 ± 39.3 | 0,815 |
| **Sys BP [mmHG]** | 120.0 ± 12.0 | 122.9 ± 17.8 | 0,468 |
| **Sys Perc [%]** | 77.3 ± 25.9 | 75.7 ± 30.2 | 0,702 |
| **Sys BP SDS** | 1.0 ± 1.3 | 1.2 ± 1.6 | 0,71 |
| **Diast. BP [mmHg]** | 61.5 ± 10.6 | 66.7 ± 11.0 | 0.056 |
| **Diast Perc [%]** | 46.6 ± 25.4 | 59.9 ± 25.9* | 0,032 |
| **Diast. BP SDS** | -0.1 ± 1 | 0.3 ± 0.9 | 0.057 |
| **Heart Rate [bpm]** | 99.5 ± 4.9 | 99,9 ± 5.0 | 0,696 |
| **SDNN [ms]** | 108.1 ± 30.8 | 105.2 ± 31.5 | 0,937 |
| **RMSSD [ms]** | 22.6 ± 7.9 | 22.7 ± 6.9 | 0,345 |
| ***TP 24h [ms^2^]*** | 2507.8 ± 1661.4 | 2217.3 ± 955.6 | 0,537 |

**3.**

The question if methylphenidate treatment is the cause of inappropriate sinus tachycardia in children with attention deficit disorder is very important and our first hypothesis. That is the reason why we test the kids with and without methylphenidate in a second Holter ECG. To our big surprise that is not the case as shown in table 3 (heart rate : 91.3 ± 7.5 bpm versus 91.9 ± 8.0 bpm). This is a very good information for millions of children who are treated with methylphenidate. To the best of our knowledge, we are the first who proof this hypothesis as everybody thinks that methylphenidate increases the heart rate as shown in the studies with single measurement after methylphenidate intake.

**4 + 5.**

We change the paragraph study Limitations:

Study Limitation

We must divide this large patient group into many (not mutually disclosing) groups according diagnosis, height, body mass index and blood pressure. This methodological problem was part of the reviewing process. We include a binary logistic regression analysis which considers all diagnoses of the patients we examined and give us the opportunity to declare odds ratios for the risk of IST.

This is a retrospective analysis including Holter ECG’s from 2005. During these 15 years, we realize the impact of nutrition and omega-3-fatty acid supplementation on heart rate variability. The multiple individual therapeutic decisions cannot be a part of this publication. We focus on methylphenidate therapy in ADHD and omega-3-fatty acid supplementation in autonomic dysfunction. As pediatric cardiologists, nearly all children had in part multiple ECG and echocardiographies that are not analyzed for this publication. The reader may be certain that none of the patients have an unrecognized heart disease.

We collect the raw data and we were able to transfer new methodological knowledge to older data sets. Today, we emphasize the importance of the mean heart rate, especially when it comes to risk assessment of cardiovascular endpoints. Heart rate significantly influences HRV due to both physiological and mathematical reasons. Moreover, physicians understand the heart rate much better than the sometimes-complicated method of HRV analysis. We try to sensitize pediatricians to the problem of heart rate regulation like inappropriate sinus tachycardia and offer an easily understandable cut off 95 bpm in adolescence.

However, the retrospective nature of the study – especially regarding omega-3-fatty acid supplementation – is a strong risk factor for “regression to the mean” as confounding mechanism. Moreover, a selection bias in children who got omega-3-fatty acid supplementation lies on the fact that their parents self-select themselves for the treatment, prove a higher motivation for the therapy and remain susceptible to a placebo effect. This means that the results of our analysis are hypothesis generating and deserve further exploration in a randomized control study.

Yours sincerely

Prof. Dr. Reiner Buchhorn and Dr. Christian Willaschek

1.	**Still AM, Raatikainen P, Ylitalo A, Kauma H, Ikäheimo M, Antero Kesäniemi Y, and Huikuri HV**. Prevalence, characteristics and natural course of inappropriate sinus tachycardia. *Europace* 7: 104-112, 2005.
